# Cancer Subtype Recognition Based on Laplacian Rank Constrained Multiview Clustering

**DOI:** 10.3390/genes12040526

**Published:** 2021-04-03

**Authors:** Shuguang Ge, Xuesong Wang, Yuhu Cheng, Jian Liu

**Affiliations:** School of Information and Control Engineering, China University of Mining and Technology, Xuzhou 221116, China; gesgcumt17@163.com (S.G.); wangxuesongcumt@163.com (X.W.); chengyuhu@163.com (Y.C.)

**Keywords:** cancer subtype recognition, Laplacian Rank Constrained, multiview clustering, graph learning

## Abstract

Integrating multigenomic data to recognize cancer subtype is an important task in bioinformatics. In recent years, some multiview clustering algorithms have been proposed and applied to identify cancer subtype. However, these clustering algorithms ignore that each data contributes differently to the clustering results during the fusion process, and they require additional clustering steps to generate the final labels. In this paper, a new one-step method for cancer subtype recognition based on graph learning framework is designed, called Laplacian Rank Constrained Multiview Clustering (LRCMC). LRCMC first forms a graph for a single biological data to reveal the relationship between data points and uses affinity matrix to encode the graph structure. Then, it adds weights to measure the contribution of each graph and finally merges these individual graphs into a consensus graph. In addition, LRCMC constructs the adaptive neighbors to adjust the similarity of sample points, and it uses the rank constraint on the Laplacian matrix to ensure that each graph structure has the same connected components. Experiments on several benchmark datasets and The Cancer Genome Atlas (TCGA) datasets have demonstrated the effectiveness of the proposed algorithm comparing to the state-of-the-art methods.

## 1. Introduction

Tumor is a malignant heterogeneous disease caused by changes in cellular components at the levels of expression, epigenetics, transcription and proteomics. The heterogeneity will be reflected in that the same cancer will produce the subtypes with different phenotypes, which will affect the clinical treatment and prognosis [1,2]. With the development and maturity of new generation sequencing technologies, large amounts of biological data are collected in public databases that are easily accessible to researchers [3]. For example, The Cancer Genome Atlas (TCGA), a landmark cancer genomics project, stores information on biological processes such as mRNA expression data, DNA methylation data, miRNA expression data and mutation data for more than 30 cancers and thousands of cancer patients [4]. Therefore, in order to solve the problem of cancer subtype recognition, building a multiview clustering model that makes full use of biological information plays a significant role.

In order to implement the task of clustering, scholars initially focus on dimensionality reduction, matrix decomposition and linear regression technologies. They all use different strategies to project high-dimensional data into low-dimensional feature space, and then achieve clustering by k-means [5,6,7,8,9,10]. For example, an effective classical method, iCluster [5], builds a Gaussian latent variable model and its modified version, iClusterPlus [6], considers different variable types following different linear probabilistic relationships to build a regression model. Both of them achieve a low-dimensional space with the combination of different biological characteristics. The other method, Pattern Fusion Analysis (PFA) [10], first uses an improved Principal Component Analysis (PCA) to find out a low-dimensional matrix of each sample, and then uses an adaptive alignment algorithm to build a fused low-dimensional feature space. However, these methods may further dilute the already low signal-to-noise ratio and increase the noise pollution to the results. Considering that the sample (patient) size of the biological data is much smaller than the feature (gene) size, some graph-based learning methods for cancer subtype recognition are designed [11,12,13,14,15,16,17]. These methods use the sample points to quickly construct the similarity graph, which can be converted into the problem of spectral clustering. For example, a widely mentioned algorithm, Similarity Network Fusion (SNF) [11], constructs the global and local similar networks for each data, and then integrates them into the final similar network based on the strategy of information propagation to dilute low similarity and enhance high similarity. Inspired by SNF, Ma et al. provided Affinity Network Fusion (ANF) [12], which constructs patients’ k-nearest neighbor similar network for each data type, and then fuses these networks based on the random walk method. In addition, Yu et al. proposed Multiview Clustering using Manifold Optimization (MVCMO) [17], and solved the problem of spectral clustering optimization by using the line search method on Stiefel manifold space.

However, most existing graph-based multiview clustering methods separate the data clustering process from graph learning process [18,19]. In some methods, the construction of the graph is independent of the clustering task, resulting in its performance being highly dependent on the predefined graph. Recently, some adaptive graph learning methods using a rank constraint on the Laplacian matrix have been able to directly reveal the clustering structure, which makes the graph construction closely related to the clustering task [20,21,22,23,24]. In addition, the similarity between sample points may commonly behave differently in different views in the process of graph fusion. Some existing algorithms simply take the average of the affinity graph of multiple views to represent the result of the fusion graph [25,26]. Therefore, the rich heterogeneous information is not fully utilized.

To sum up, we designed a graph-based multiview clustering algorithm, called Laplacian Rank Constrained Multiview Clustering (LRCMC). Firstly, the Laplacian Rank Constraint (LRC) algorithm [27] is used to simultaneously find the affinity graph and the embedding matrix in each view to ensure that the graph structure is on the same connected components. Then, based on the method of Nie et al. [24], we use LRC method to obtain the consistent graph, whose connected components are the same as the affinity graph of each view. Finally, the clustering structure is obtained. In the process of graph fusion, the inverse distance weighting scheme is employed to design different weights for each view’s affinity graph [24], so as to adjust the structure of the consistent graph more effectively. Moreover, the processes of graph learning, graph fusion and clustering are coupled into an optimization problem to update the more accurate consistent graph and improve the results of the clustering. In order to evaluate the effectiveness of the proposed method, experiments were carried out on four benchmark datasets and four TCGA datasets. Four start-of-the-art methods were used for comparison. The values of Accuracy (ACC), Normalized Mutual Information (NMI) and Purity on benchmark datasets, which are commonly used metrics in clustering analysis, and the *p* value obtained from survival analysis on the TCGA dataset can all show that the proposed LRCMC approach achieves considerable improvement over the state-of-the-art baseline methods. In the analysis of the Glioblastoma Multiforme (GBM) subtypes, we found these clusters have biological significance, e.g., the Proneural subtype granted by G-CIMP phenotype has a better survival advantage. The source code and datasets can be found in the Supplementary File 1.

## 2. Methods

**Figure 1:**
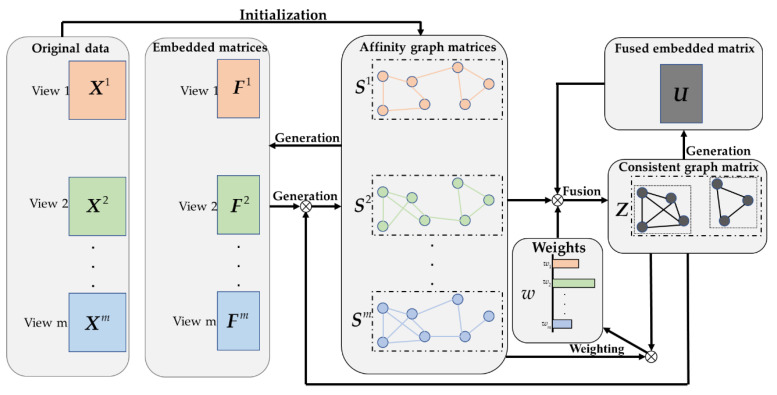
The flow chart of Laplacian Rank Constrained Multiview Clustering.

The overall flow of LRCMC is shown in Figure 1. Specifically, given a set of omics data with *m* views $X^{1},\ldots ,X^{m}$, a set of affinity graph matrices $S^{1},\ldots ,S^{m}$ are constructed, respectively, according to $X^{1},\ldots ,X^{m}$. It should be emphasized that the process of learning the affinity matrix in LRCMC is different from most multiview clustering algorithms. $S^{1},\ldots ,S^{m}$ are not calculated directly from the original matrix, but are constructed, respectively, from a set of embedding matrices $F^{1},\ldots ,F^{m}$ by the LRC method. Therefore, each affinity graph matrix is constrained to the same connected components, which ensures that each affinity graph has a similar structure before the fusion process. Then, the proposed fusion method is applied to the affinity graph matrices of all views in order to learn a consistent graph matrix ***Z***. Simultaneously, each view is automatically assigned a different weight $w_{1},\ldots ,w_{m}$ to represent its contribution to ***Z*** during the fusion process. Finally, the learned consistent graph matrix ***Z*** is used to optimize the affinity graph matrix for each view. The LRC method is also imposed to constrain that the number of connected components in the ***Z*** is equal to the required number of clusters *c* by constructing the fusion embedded matrix ***U***. Our LRCMC improves the affinity matrix of each view, builds a fused consistent graph matrix and obtains clustering results simultaneously.

### 2.1. Construction of Affinity Graph Based on LRC

Given a single biological data $X^{v}=\left\{x_{1}^{v},\ldots ,x_{n}^{v}\right\}\in R^{d_{v}\times n}$ denotes the *v*-th view data with *d_v_* features, where *n* is the number of data points. $S^{v}\in R^{n\times n}$ represents the similar relationship between the sample points in the graph learning framework. The smaller the distance between a pair of vertices in the graph is, the greater the similarity between the pair of vertices will be, the greater the corresponding weight will be, and vice versa. Based on the manifold structure of graph, the most traditional way to build $S^{v}$ is by generating a k-nearest neighbor graph for it. A pair of vertices are considered connected if they are near neighbors. There are other effective strategies to design more accurate affinity graph $S^{v}$, e.g., smooth representation [28], Gaussian kernel for similarity learning [29], etc. For the purpose of clustering, if the sample points can be assigned to the *c* categories, the obtained $S^{v}$ should contain exact *c* connected components. Based on the following Theorem 1, $S^{v}$ can be realized.

**Theorem** **1.** *Multiplicity c of zero eigenvalues of Laplacian matrix*$L^{v}$*is equal to the number of connected components of its similarity matrix*$S^{v}$.

When all the elements in $S^{v}$ satisfy the non-negative condition, its Laplacian matrix $L^{v}$ has the above property [30,31].

Theorem 1 means if $\sum_{i=1}^{c}\lambda_{i}=0$, where $\lambda_{i}$ is *i*-th smallest eigenvalue of $L^{v}$, the data points on $S^{v}$ have been ideally assigned to *c* categories [32], Laplacian rank meets the constraint condition $\mathrm{rank}\left(L^{v}\right)=n-c$. Therefore, based on the Ky Fan’s theorem [33], we can minimize $\sum_{i=1}^{c}\lambda_{i}$ approximately meeting the requirement of Theorem 1. The objective function is written as:

(1) $$\sum_{i=1}^{c}\lambda_{i}=\underset{F^{v}\in R^{n\times c},{\left(F^{v}\right)}^{T}F^{v}=I}{\min}\mathrm{Tr}\left({\left(F^{v}\right)}^{T}L^{v}F^{v}\right)$$

where $F^{v}\in R^{n\times c}$ is obtained by the *c* eigenvectors of $L^{v}$ corresponding to the *c* smallest eigenvalues. Tr(.) denotes the trace operator, $L^{v}=D^{v}-\left[\left({\left(S^{v}\right)}^{T}+S^{v}\right)/2\right]$ is the Laplacian matrix, $D^{v}$ is a diagonal matrix and its elements are column sums of $\left[\left({\left(S^{v}\right)}^{T}+S^{v}\right)/2\right]$.

However, the solution to $F^{v}$ in Equation (1) is actually to solve trivial solution to $S^{v}$. Therefore, a $\ell_{2}$-norm regularization term is employed to obtain smooth $S^{v}$ and each column of $S^{v}$ satisfies $1^{T}s_{j}^{v}=1$, where $s_{j}^{v}$ is the *j*th column of $S^{v}$ [21]. Finally, we can obtain the objective function related to $F^{v}$ and $S^{v}$ simultaneously:

(2) $$\begin{array}{l} \underset{F^{v},S^{v}}{\min}2\mathrm{Tr}\left({\left(F^{v}\right)}^{T}L^{v}F^{v}\right)+\alpha {\left\|S^{v}\right\|}_{F}^{2}\\ s.t.{\left(F^{v}\right)}^{T}F^{v}=I,\forall j,1^{T}s_{j}^{v}=1,s_{j}^{v}\geq 0,s_{jj}^{v}=0 \end{array}$$

where α is the regularization parameter.

A set of the affinity graph matrices $S^{1},\ldots ,S^{m}$ and the embedded matrices $F^{1},\ldots ,F^{m}$ are obtained through Equation (2) without the participation of the original data. However, these affinity matrices are unrelated; if they are simply stacked together for clustering, the graphs will be badly damaged and the algorithm performance will degrade. Therefore, we need to introduce a graph fusion strategy to construct a consistent graph matrix with the unified connected components.

### 2.2. Graph Fusion with LRC

Integrating these basic graphs to form the fused affinity graph $Z\in R^{n\times n}$, two intuitive points should be considered: (1) The designed graph $S^{v}$ for each view can be considered as the consistent graph ***Z*** with noise representation and outlier interference. (2) $S^{v}$ closer to ***Z*** should be given greater weight to reduce the perturbation of the low-quality graphs on the fusion graph. In this way, ***Z*** can accurately capture the true similarity hidden in the multiview data. Therefore, we employed the proposed method of Nie et al. [24] to optimize ***Z*** as follows:

(3) $$\underset{Z}{\min}\sum_{v=1}^{m}w_{v}{\left\|Z-S^{v}\right\|}_{F}^{2}$$

where $w_{v}$ is the weight of the single affinity graph $S^{v}$. The inverse distance weighting scheme is designed to calculate $w_{v}$. The Lagrange function of Equation (3) can be written as:

(4) $$\underset{Z}{\min}\sum_{v=1}^{m}w_{v}{\left\|Z-S^{v}\right\|}_{F}^{2}+\varsigma \left(\Lambda ,Z\right)$$

where Λ is the Lagrange multiplier, ς(Λ,Z) is the formal term derived from constraint condition. Taking the derivative of Equation (4) w.r.t ***Z*** and setting the derivative to zero, we can obtain:

(5) $$\sum_{v=1}^{m}w_{v}\frac{\partial {\left\|Z-S^{v}\right\|}_{F}^{2}}{\partial Z}+\frac{\partial \varsigma \left(\Lambda ,Z\right)}{\partial Z}=0$$

where $w_{v}$ is given as follows:

(6) $$w_{v}=\frac{1}{2\sqrt{{\left\|Z-S^{v}\right\|}_{F}^{2}}}$$

Here, a set of weights $w_{1},\ldots ,w_{m}$ and a consistent graph matrix ***Z*** are obtained from Equation (3). In order to make the learned ***Z*** also have *c* connected components for clustering, the LRC term is added to Equation (3) according to Theorem 1 and Ky Fan’s Theorem. The objective function is as follows:

(7) $$\begin{array}{l} \underset{Z}{\min}\sum_{v=1}^{m}w_{v}{\left\|Z-S^{v}\right\|}_{F}^{2}+2\beta \mathrm{Tr}\left(U^{T}L_{Z}U\right)\\ s.t.\;U^{T}U=I,\forall j,1^{T}z_{j}=1,z_{j}\geq 0,z_{jj}=0 \end{array}$$

where $U\in R^{n\times c}$ is obtained by the *c* eigenvectors of $L_{Z}$ corresponding to the *c* smallest eigenvalues, i.e., the embedded matrix corresponding to ***Z***. $L_{Z}=D_{Z}-\left[\left(Z^{T}+Z\right)/2\right]$ is the Laplacian matrix, $D_{Z}$ is a diagonal matrix and its elements are column sums of $\left[\left(Z^{T}+Z\right)/2\right]$. β is the regularization parameter.

### 2.3. LRCMC Algorithm

As described in Section 2.1 and Section 2.2, the LRC operation is used to guarantee the structures of $S^{1},\ldots ,S^{m}$ and ***Z***. Therefore, we can combine Equations (2) and (7) into a final objective function, i.e., the proposed Laplacian Rank Constrained Multiview Clustering (LRCMC). It is represented as:

(8) $$\begin{array}{l} \underset{F^{v},S^{v},w_{v},Z,U}{\min}\sum_{v=1}^{m}2\mathrm{Tr}({\left(F^{v}\right)}^{T}L^{v}F^{v})+\alpha {\left\|S^{v}\right\|}_{F}^{2}+w_{v}{\left\|Z-S^{v}\right\|}_{F}^{2}+2\beta \mathrm{Tr}\left(U^{T}L_{Z}U\right)\\ s.t.\;(F^{v}{)}^{T}F^{v}=I,U^{T}U=I,\\ \forall j,1^{T}s_{j}^{v}=1,s_{j}^{v}\geq 0,s_{jj}^{v}=0,1^{T}z_{j}=1,z_{j}\geq 0,z_{jj}=0 \end{array}$$

Here, we complete the tasks of graph construction, graph fusion and clustering in one step through the integrated model. In this way, the learning of $S^{1},\ldots ,S^{m}$ and ***Z*** can help each other embedded in a joint coupling problem. The objective function Equation (8) enjoys the following properties:

- Our method can effectively learn a set of affinity graph matrices with *c* connected components, instead of most multiview clustering methods requiring predefined graphs;
- In the graph fusion process, we assign the weight to each view to represent their contribution to the consistent graph ***Z***, rather than simply superimposing then together;
- We use LRC to constantly adjust the structures of $S^{1},\ldots ,S^{m}$ and ***Z***, and at the same time complete the task of clustering.

### 2.4. Optimization Algorithm of LRCMC

Obviously, since the variables in Equation (8) are coupled to each other, we use alternating iterative method and Augmented Lagrange Multiplier (ALM) scheme to solve $S^{1},\ldots ,S^{m}$, $F^{1},\ldots ,F^{m}$, $w_{1},\ldots ,w_{m}$, ***Z***, ***U***. The specific solution process is as follows:

1. Fix $F^{1},\ldots ,F^{m}$, $w_{1},\ldots ,w_{m}$, ***Z*** and ***U***, solve $S^{1},\ldots ,S^{m}$;

The Equation (8) becomes:

(9) $$\begin{array}{l} \underset{S^{v}}{\min}\sum_{v=1}^{m}2\mathrm{Tr}({\left(F^{v}\right)}^{T}L^{v}F^{v})+\alpha {\left\|S^{v}\right\|}_{F}^{2}+w_{v}{\left\|Z-S^{v}\right\|}_{F}^{2}\\ s.t.\;\forall j,1^{T}s_{j}^{v}=1,s_{j}^{v}\geq 0,s_{jj}^{v}=0 \end{array}$$

Due to $\mathrm{Tr}({\left(F^{v}\right)}^{T}L^{v}F^{v})=\frac{1}{2}\sum_{i,j}{\left\|f_{i}^{v}-f_{j}^{v}\right\|}_{2}^{2}s_{ij}^{v}$, where $f_{i}^{v}$ and $f_{j}^{v}$ denote the *i*-th and *j*-th column of $F^{v}$, respectively, $s_{ij}^{v}$ denotes the (*i, j*)th element of $S^{v}$, the Equation (9) can be written in vector form as:

(10) $$\begin{array}{l} \underset{s_{j}^{v}}{\min}\sum_{i=1}^{n}{\left\|f_{i}^{v}-f_{j}^{v}\right\|}_{2}^{2}s_{ij}^{v}+\alpha {\left\|s_{j}^{v}\right\|}_{2}^{2}+w_{v}{\left\|z_{j}-s_{j}^{v}\right\|}_{2}^{2}\\ s.t.\;s_{jj}^{v}=0,s_{j}^{v}\geq 0,1^{T}s_{j}^{v}=1 \end{array}$$

Denote $p_{ij}^{v}={\left\|f_{i}^{v}-f_{j}^{v}\right\|}_{2}^{2}$, Equation (10) is obviously written as:

(11) $$\begin{array}{l} \underset{s_{j}^{v}}{\min}\sum_{i=1}^{n}p_{ij}^{v}s_{ij}^{v}+\alpha {\left\|s_{ij}^{v}\right\|}_{2}^{2}+w_{v}{\left\|z_{ij}-s_{ij}^{v}\right\|}_{2}^{2}\\ s.t.\;s_{jj}^{v}=0,s_{j}^{v}\geq 0,1^{T}s_{j}^{v}=1 \end{array}$$

Then, the Equation (11) can be written as:

(12) $$\underset{s_{j}^{v}}{\min}{\left\|s_{j}^{v}+\frac{p_{j}^{v}/2-z_{j}}{\alpha +w_{v}}\right\|}_{2}^{2}$$

Therefore, the Lagrangian function of Equation (12) combined with its constraints can be defined as:

(13) $$\ell \left(s_{j}^{v},\eta ,\varphi \right)=\frac{1}{2}{\left\|s_{j}^{v}+\frac{p_{j}^{v}/2-z_{j}}{\alpha +w_{v}}\right\|}_{2}^{2}+\eta \left(1^{T}s_{j}^{v}-1\right)+\varphi^{T}s_{j}^{v}$$

where η is the Lagrangian coefficient scalar and φ is the Lagrangian coefficient vector. Based on the Karush-Kuhn-Tucker (KKT) condition [34], the optimal solution of $s_{j}^{v}$ can be estimated as:

(14) $$s_{j}^{v}={\left(\frac{p_{j}^{v}/2-z_{j}}{\alpha +w_{v}}+\eta \right)}_{+}$$

The study in [35] found that sparse representation is robust to noise and outliers. In order to obtain the sparse affinity graph $S^{v}$, we can find the *k* nonzero adaptive neighbors for $s_{j}^{v}$ to satisfy $s_{jk}^{v}>0$ and $s_{j,k+1}^{v}=0$. Denote $\alpha +w_{v}=\delta$, then, we arrive at:

(15) $$-\frac{p_{jk}^{v}}{2}+w_{v}z_{jk}+\delta \eta >0,-\frac{p_{j,k+1}^{v}}{2}+w_{v}z_{j,k+1}+\delta \eta \leq 0$$

Moreover, according to Equation (15) and the constraint condition $1^{T}s_{j}^{v}-1=0$, η is given:

(16) $$\eta =\frac{1}{k}\left(1+\frac{w_{v}}{\delta }+\frac{\sum_{l=1}^{k}p_{jl}^{v}}{2\delta }\right)$$

Therefore, according to Equations (15) and (16), the range of δ is obtained as follows:

(17) $$\left\{ \begin{array}{l} \delta >\frac{kp_{jk}^{v}-2kw_{v}z_{jk}-2w_{v}-\sum_{l=1}^{k}p_{jl}^{v}}{2}\\ \delta \leq \frac{kp_{j,k+1}^{v}-2kw_{v}z_{j,k+1}-2w_{v}-\sum_{l=1}^{k}p_{jl}^{v}}{2} \end{array}\right.$$

Then, the parameter δ can be set as:

(18) $$\delta =\frac{kp_{j,k+1}^{v}-2kw_{v}z_{j,k+1}-2w_{v}-\sum_{l=1}^{k}p_{jl}^{v}}{2}$$

Finally, according to Equations (15), (16) and (18), the optimal solution of $s_{j}^{v}$ in $s_{ij}^{v}$ is represented as:

(19) $$s_{ij}^{v}=\left\{ \begin{array}{l} \frac{p_{j,k+1}^{v}-p_{ij}^{v}+2w_{v}z_{ij}-2w_{v}z_{j,k+1}}{kp_{j,k+1}^{v}-\sum_{l=1}^{k}p_{jl}^{v}-2kw_{v}z_{j,k+1}+2\sum_{l=1}^{k}w_{v}z_{jl}}\;j\leq k\\ 0\;\;\;\;\;\;\;\;\;\;\;j>k \end{array}\right.$$

2. Fix $S^{1},\ldots ,S^{m}$, $w_{1},\ldots ,w_{m}$, ***Z*** and ***U***, solve $F^{1},\ldots ,F^{m}$;

Updating $F^{v}$ in Equation (8) is converted to Equation (2). Therefore, $F^{v}$ is updated from Equation (1) in Section 2.1.

3. Fix $S^{1},\ldots ,S^{m}$, $F^{1},\ldots ,F^{m}$, ***Z*** and ***U***, solve $w_{1},\ldots ,w_{m}$;

Updating $w_{v}$ in Equation (8) is equivalent to Equation (3). Therefore, $w_{v}$ is updated from Equation (6) in Section 2.2.

4. Fix $S^{1},\ldots ,S^{m}$, $F^{1},\ldots ,F^{m}$, $w_{1},\ldots ,w_{m}$ and ***U***, solve ***Z***.;

Updating Z in Equation (8) is converted to Equation (7). Due to $\mathrm{Tr}(U^{T}L_{Z}U)=\frac{1}{2}\sum_{i,j}{\left\|u_{i}-u_{j}\right\|}_{2}^{2}z_{ij}$, where $u_{i}$ and $u_{j}$ denote the i-th and j-th column of ***U***, $z_{ij}$ denotes the (*i*, *j*)th element of ***Z***, Equation (7) yields:

(20) $$\begin{array}{l} \underset{z_{j}}{\min}\sum_{v=1}^{m}\sum_{i}^{n}\left\{w_{v}{\left\|z_{j}-s_{j}^{v}\right\|}_{2}^{2}+\beta {\left\|u_{i}-u_{j}\right\|}_{2}^{2}z_{ij}\right\}\\ s.t.\;1^{T}z_{j}=1,z_{j}\geq 0,z_{jj}=0 \end{array}$$

Denote $q_{ij}={\left\|u_{i}-u_{j}\right\|}_{2}^{2}$, we have:

(21) $$\begin{array}{l} \underset{z_{j}}{\min}\sum_{v=1}^{m}{\left\|z_{j}-s_{j}^{v}+\frac{\beta }{2mw_{v}}q_{j}\right\|}_{2}^{2}\\ s.t.\;1^{T}z_{j}=1,z_{j}\geq 0,z_{jj}=0 \end{array}$$

Based on the Karush–Kuhn–Tucker (KKT) condition [34], the closed form solution of $z_{j}$ can be estimated as:

(22) $$z_{j}={\left(s_{j}^{v}-\frac{\beta }{2mw_{v}}q_{j}+\eta \right)}_{+}$$

Equation (22) can be solved by an efficient optimization method proposed in [35].

5. Fix $S^{1},\ldots ,S^{m}$, $F^{1},\ldots ,F^{m}$, $w_{1},\ldots ,w_{m}$ and ***Z***, solve ***U***.

According to the method of finding $F^{v}$, ***U*** is obtained as follows:

(23) $$\underset{U\in R^{n\times c},U^{T}U=I}{\min}\mathrm{Tr}\left(U^{T}L_{Z}U\right)$$

The final solution of ***U*** is the *c* eigenvectors of $L_{Z}$ corresponding to the *c* smallest eigenvalues.

## 3. Experiments’ Results

In order to verify effectiveness of LRCMC in cancer subtype recognition, LRCMC was compared with four state-of-the-art clustering algorithms, i.e., ANF [12], SNF [11], PFA [10] and MVCMO [17]. Since biological omics data are not labeled, we first downloaded four widely used benchmark datasets containing real labels, i.e., 3-source, Calt-7, MSRC, WebKB, to verify that proposed LRCMC can achieve good clustering effect. Furthermore, we applied LRCMC to the datasets downloaded and preprocessed by Wang et al. [11] from TCGA. The datasets contain four types of cancer, i.e., GBM, Breast Invasive Carcinoma (BIC), Lung Squamous Cell Carcinoma (LSCC) and Colon Adenocarcinoma (COAD).

### 3.1. Comparison Experiments on Benchmark Datasets

The benchmark datasets are described as follows:

- 3-source [20]: It contains 169 news that were reported by three news magazines, i.e., BBC, Reuters, and The Guardian. There are six different thematic labels for each news;
- Calt-7 [36]: The object recognition dataset is drawn from the Caltech101 dataset to screen 7 widely used classes, i.e., faces, motorbikes, dollar bill, Garfield, stop sign, and Windsor chair. Each class has 1474 images. Each image is described by 6 features, i.e., GABOR, wavelet moment (WM), CENT, HOG, GIST and LBP;
- MSRC [37]: The scene recognition dataset contains 7 classes of aircraft, car, bicycle, cow, faces, tree, and building. Each image is described by 5 features, i.e., color moment (CMT), HOG, LBP, CENT, GIST;
- WebKB [20]: It collects 203 web pages in 4 classes from the University’s Computer science department. Each page has 3 features, i.e., the content of the page, the anchor text of the hyperlink, and the text description in the title.

Table 1 is an overview of these datasets, where *n*, *m*, and *c* describe the number of samples, views, and classes for each dataset, respectively, *d_v_* denotes the *i*-th feature of these datasets.

Three commonly used evaluation metrics, i.e., Accuracy (ACC), Normalized Mutual Information (NMI) and Purity, are used to quantitatively measure the clustering performance of the algorithms. The metrics compare the resulting labels with the real labels provided by the dataset. The larger the value obtained, the better the clustering results. To ensure the fairness of the comparison experiments, each algorithm was run 10 times to reduce the impact of randomness. The mean and standard deviation of the obtained metrics were calculated. In addition, the neighbor *k* required by ANF, SNF, MVCMO and LRCMC was set within the range of [5,50], and other parameters were specified as the default values provided by the authors. Only one parameter β needs to be set in our LRCMC algorithm, which is caused by the introduction of LRC. In order to achieve rapid convergence of Algorithm 1, we adopt a dynamic parameter updating method proposed by Nie et al. [23]. β is set in the range of [1,30]. If the number of connected components of ***Z*** is greater than *c*, we will shrink β (β=β/2). On the contrary, if less than *c*, we will increase β (β=2×β) until finding the right components for ***Z***. Table 2 shows the final evaluation metrics obtained by these algorithms in the four datasets. It is obvious that LRCMC achieves better clustering performance in the multiview clustering task than the other methods.
**Algorithm 1.** LRCMC algorithm**Input:** Original data $X^{1},\ldots ,X^{m}$ with *m* views, the number of clusters *c*, the number of neighbors *k*, the regularization parameter β. **Output:** The learned consensus matrix ***Z***. Initialize the affinity matrices $S^{1},\ldots ,S^{m}$ for each view by solving the following problem: $\underset{s_{j}^{v}}{\min}\sum_{i=1}^{n}{\left\|x_{i}-x_{j}\right\|}_{2}^{2}s_{ij}^{v}+\alpha {\left\|s_{j}^{v}\right\|}_{2}^{2}$; Initialize the embedded matrices $F^{1},\ldots ,F^{m}$ for each view by using Equation (1); Initialize the weights $w_{1},\ldots ,w_{m}$ for each view by $w_{v}=1/m$; Initialize ***Z*** by connecting $S^{1},\ldots ,S^{m}$ with $w_{1},\ldots ,w_{m}$; Initialize the fused embedded matrix ***U*** by using Equation (23); **Repeat** Fix $F^{1},\ldots ,F^{m}$, $w_{1},\ldots ,w_{m}$, ***Z*** and ***U***, update $S^{1},\ldots ,S^{m}$ by using Equation (19); Fix $S^{1},\ldots ,S^{m}$, $w_{1},\ldots ,w_{m}$, ***Z*** and ***U***, update $F^{1},\ldots ,F^{m}$ by using Equation (1); Fix $S^{1},\ldots ,S^{m}$, $F^{1},\ldots ,F^{m}$, ***Z*** and ***U***, update $w_{1},\ldots ,w_{m}$ by using Equation (6); Fix $S^{1},\ldots ,S^{m}$, $F^{1},\ldots ,F^{m}$, $w_{1},\ldots ,w_{m}$ and ***U***, update ***Z*** by using Equation (22); Fix $S^{1},\ldots ,S^{m}$, $F^{1},\ldots ,F^{m}$, $w_{1},\ldots ,w_{m}$ and ***Z***. update ***U*** by using Equation (23); **Until** Satisfy Theorem 1 or the maximum iteration reached. The learned consensus matrix ***Z*** with exact *c* connected components, which are the final clusters.

### 3.2. Comparison Experiments on TCGA Datasets

To demonstrate the effectiveness of LRCMC in identifying cancer subtype, the designed LRCMC was applied to four cancer omics datasets, i.e., GBM, BIC, LSCC, and COAD. Each cancer subtype contains three types of expression data from different platforms, i.e., mRNA expression data, DNA methylation data and miRNA expression data. Table 3 shows the number of samples (patients) and features (genes) held by each cancer subtype.

**Figure 2:**
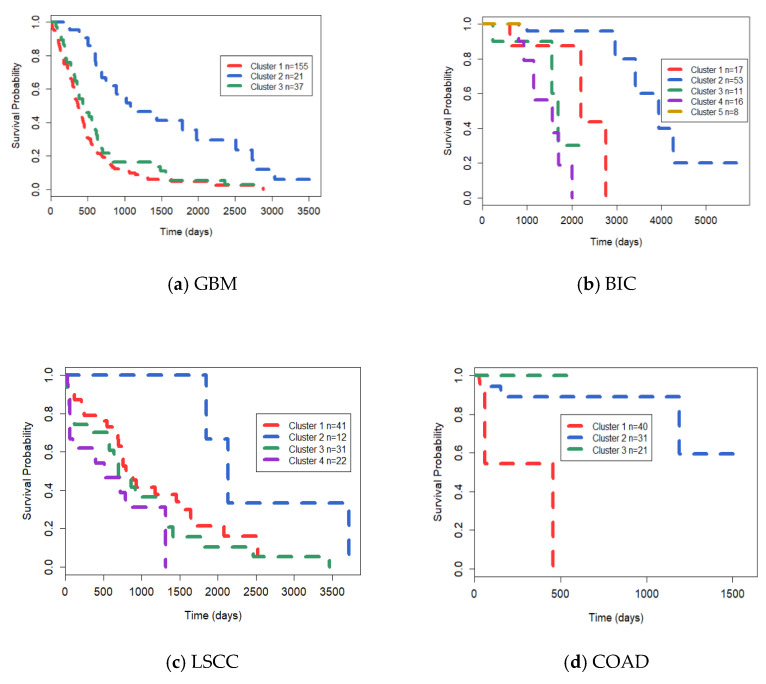
The Kaplan–Meier survival curves of (**a**): Glioblastoma Multiforme (GBM), (**b**): Breast Invasive Carcinoma (BIC), (**c**): Lung Squamous Cell Carcinoma (LSCC) and (**d**): Colon Adenocarcinoma (COAD), respectively.

To ensure that the identified cancer labels conform to the true clinical diagnosis, we specified that the number of samples in each cluster should be at least 3. We used the number of subtypes of GBM, BIC, LSCC, and COAD specified by Wang et al., which were 3, 5, 4 and 3, respectively. Then, the *p* values based on Cox log-rank model were used to evaluate the clustering results of these algorithms in survival analysis [38]. If the *p* value is smaller, the survival rate between different groups is more significant and the difference is greater, which means the cluster is considered to have different characteristics of the underlying cancer subtypes. Cancer survival curves can also represent heterogeneity between different subtypes. As shown in Table 4, LRCMC obtained the best *p* value in BIC, GBM, KRCCC and COAD. Other algorithms also had good results in specific datasets, but they were all lower than our algorithm. Therefore, we believe that LRCMC is significantly advantageous in the topic of cancer subtype recognition. Figure 2 shows the Kaplan–Meier survival analysis curves of the four cancers. Each curve depicts trends in the survival time of each cancer cluster and the number of samples for each cluster is also shown in the figure.

### 3.3. Analysis on GBM Dataset

GBM is the most malignant glioma among astrocytomas. It has been studied and analyzed at the genetic level by many scholars, and specific subtypes and treatment protocols have been proposed. For example, according to the mRNA expression data, Verhaak et al. [39] reported that GBM is divided into Mesenchymal, Classical, Neural and Proneural subtypes, and the heterogeneous subtypes were also verified in somatic mutations and copy number variations (CNVs). Another study divided GBM patients into two subtypes, i.e., G-CLMP and non-G-CLMP, based on the difference of CpG Island methylator phenotype (CLMP) [40]. Table 5 shows the distribution of the cluster results obtained by LRCMC on the subtype identified by these two studies. From Table 5, there are more patients in cluster 1 than in cluster 3, and all of them are assigned to non-G-CLMP subtype—also they have four subtypes identified based on mRNA expression. The point is that the Proneural subtype in these two clusters belong to non-G-CLMP subtype. However, cluster 2, with a smaller number of patients, is almost the Proneural subtype, and also belongs to G-CLMP subtype.

**Figure 3:**
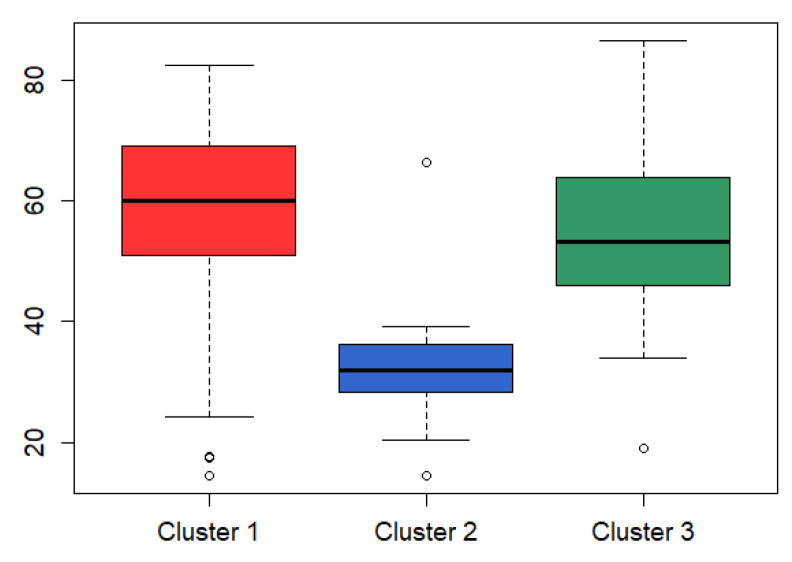
Boxplot of diagnosis age for the identified clusters. It reflects the distribution of diagnosis age in each cluster. Black bar represents the median of each cluster.

**Figure 4:**
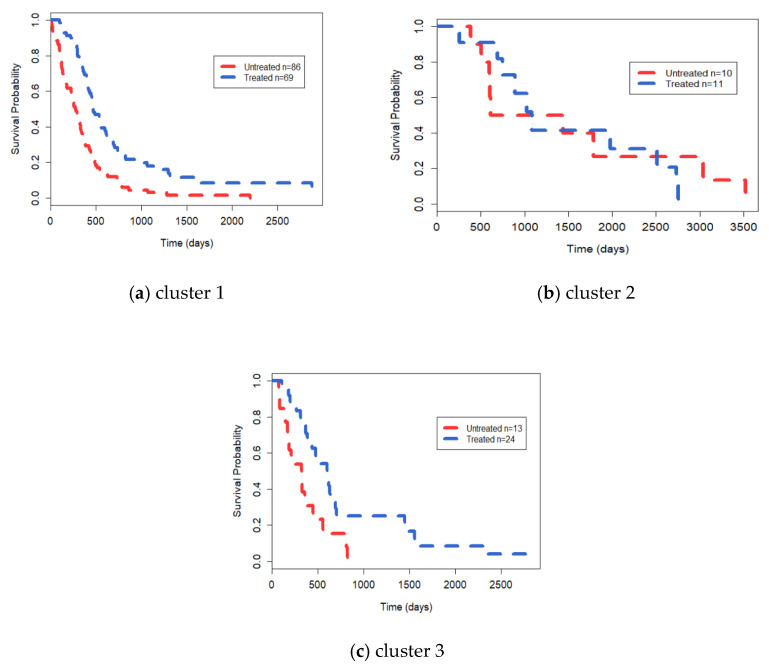
The Kaplan–Meier survival curves of the identified clusters (**a**): cluster 1, (**b**): cluster 2 and (**c**): cluster 3) of Temozolomide (TMZ) response. “Untreated” expresses the group which did not receive TMZ treatment and “Treated” expresses the group which received TMZ treatment.

To further analyze the identified clusters, we downloaded clinical data, somatic mutation data and CNV data for all patients from the cBio Cancer Genomis Portal database (http://www.cbioportal.org/ accessed on 15 December 2020). The age profiles of the three clusters (Figure 3), differential gene statistics of CNVs and mutations (Table 6), and Kaplan–Meier survival curves of Temozolomide (TMZ) (Figure 4) in GBM patients were obtained. Figure 3 shows that the diagnosis age of patients in cluster 2 with the best survival advantage is also lower than that of patients in cluster 1 and cluster 3. The genetic variant signatures associated with GBM in terms of mutation (*IDH1*) and CNVs (*CDKN2A*, *CDKN2B*, *C9orf53*, *MTAP*, *EGFR*) are significantly different in the three identified clusters. In particular, *IDH1* mutation only occurs in cluster 2, while *EGFR* amplification is 0. Then, we divided the patients within the three clusters into two groups: patients treated with TMZ and those not treated with TMZ, then we compared the drug response. TMZ is a drug that is commonly used to treat GBM, but only responds well to a subset of patients. The *p* values of survival analysis in Cox log-rank model of the three cluster comparison experiments are 2.0 × 10^−6^, 0.76 and 0.01, respectively, which indicates that TMZ treatment has no effect on the patients in cluster 2. Therefore, in summary, we can infer that the subtype belonging to G-CLMP subtype and Proneural subtype might be a potentially new subtype. This also verified by the fact that that the Proneural subtype granted by the G-CIMP phenotype proposed by Canmenron et al. has unique properties [41].

**Figure 5:**
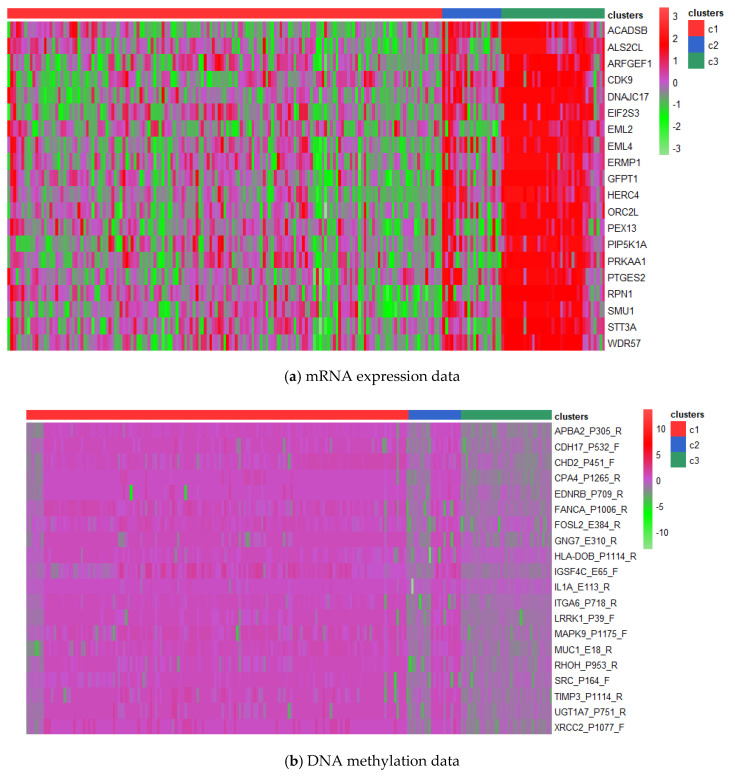
Heatmaps of differentially expressed genes in (**a**): mRNA expression data and (**b**): DNA methylation data for the identified clusters.

In addition, mRNA expression data and DNA methylation data were used to compare the differentially expressed genes in cluster 1 and 3 to look for the heterogeneity between them. We compared the genes in the two clusters using ANOVA (the lower the *p*-value, the higher the ranking). The gene differences in miRNA expression data were not significant enough (*p* values were all greater than 0.1) and were omitted from consideration. Figure 5 shows the heatmaps of the top 20 differentially expressed genes in the mRNA expression data and the DNA methylation data, respectively. It is obvious that cluster 1 and cluster 3 are different in gene expression level, and some of the genes on the heatmaps have been shown to be linked to GBM., e.g., *PRKAA1* overexpressed in Cluster 3, also known as *AMPK*, induces antitumor activity in GBM cells and has become a possible tumor control target [42]. *MUC1* overexpressed in cluster 1 is a pathogenic gene that induces GBM and can be used as a target for cellular immunotherapy [43].

Finally, we compared the three clusters with normal samples and screened for differentially expressed genes using ANOVA. We did Gene Ontology (GO: BP), KEGG pathway and Disease Ontology (DO) enrichment analysis using the top 100 differential genes in ToppGene Suite database (https://toppgene.cchmc.org/enrichment.jsp accessed on 20 December 2020). From Table 7, it is clear that the biological processes of cluster 1 are related to “epithelium development” and “cell adhesion”, while the biological processes of cluster 2 and 3 are mostly related to “protein targeting” and “protein localization”. Moreover, it is interesting to note that all three clusters are associated with anemia in DO enrichment analysis. It is possible that GBM patients treated with TMZ will develop aplastic anemia [44].

## 4. Discussion and Conclusions

Over the past decade, it has been widely recognized that the integration and mining of different types of biological data provides meaningful insights into the causes and complexity systems of cancer [45]. Now, the challenge is still how to capture the underlying structure of the sample/features from the omics data for application to a wide range of bioinformatics topics, e.g., the prediction of drug-target relationships [46], the recognition of cancer driver genes [47], finding out about genotype–epigenetic interactions [48], etc.

In this paper, our proposed LRCMC algorithm has the ability to fuse multigenomic data into the consensus graph of the exact connected components. In fact, the sample size of a given cancer is generally relatively small, so the graph learning method can quickly map the feature space into the structure of the affinity graph without the need for feature prescreening. Based on the framework of graph learning, LRCMC uses the operation of LRC to mine the structure of clustering while maintaining the graph structure. At the same time, the graph obtained by the adaptive neighbors method is sparse, so that the weak similarity relationship is even more sparse at 0, which ensures more accurate clustering results. Compared with other start-of-the-art integration clustering algorithms for cancer subtype recognition, LRCMC has the following two characteristics: (1) instead of simply treating each view equally, a wealth of heterogeneous information is taken into account to provide appropriate weight for each view; (2) the tasks of constructing the affinity matrix of each view, learning the fused matrix and clustering are completed simultaneously in a system. Furthermore, LRCMC has the following two advantages in algorithm running: (1) there is no need to spend a lot of time choosing the appropriate parameters; (2) the final consensus graph has been assigned to the given categories without adding additional base clustering algorithms. We demonstrated the power of LRCMC using four benchmark datasets and four cancer datasets. The experiments show that LRCMC has a good clustering evaluation. The cancer subtype recognition results on GBM data show that LRCMC can effectively capture cancer subtypes with specific biological characteristics based on omics data.

In addition, we must admit that LRCMC also has shortcomings and limitations. It is not suitable for binary data (somatic mutation) or categorical data (copy number states: loss/normal/gain), and has only limited application to continuous data (mRNA expression) to identify cancer subtype. It also does not have the ability to find the gene modules that affect differences in each subtype. Therefore, we will continue our efforts to improve and extend the LRCMC algorithm to explore cancer heterogeneity.

## Figures and Tables

**Table 1:** Overview of four benchmark datasets.

Dataset	*n*	*m*	*c*	*d_1_*	*d_2_*	*d_3_*	*d_4_*	*d_5_*	*d_6_*
3-source	169	3	6	3560	3631	3638	-	-	-
Calt-7	1474	6	7	48	40	254	1984	512	928
MSRC	210	5	7	48	100	256	1302	512	-
WebKB	203	3	4	1703	230	230	-	-	-

**Table 2:** The clustering performance comparison in terms of ACC, NMI and Purity on the four real datasets.

Datasets	Methods	ACC	NMI	Purity
3-source	ANF	0.4970 (0.0000)	0.2804 (0.0000)	0.5325 (0.0000)
	SNF	0.7811 (0.0000)	0.6942 (0.0000)	0.8166 (0.0000)
	PFA	0.4562 (0.0761)	0.2247 (0.0713)	0.7160 (0.0578)
	MVCMO	0.4221 (0.0123)	0.3035 (0.0128)	0.5266 (0.0118)
	LRCMC	**0.8107 (0.0000)**	**0.7218 (0.0000)**	**0.8462 (0.0000)**
Calt-7	ANF	0.6696 (0.0000)	0.6203 (0.0000)	0.8684 (0.0000)
	SNF	0.6601 (0.0000)	0.5637 (0.0000)	0.8562 (0.0000)
	PFA	-	-	-
	MVCMO	0.6654 (0.0100)	0.5179 (0.0355)	0.8464 (0.0083)
	LRCMC	**0.8548 (0.0000)**	**0.7694 (0.0000)**	**0.8921 (0.0000)**
MSRC	ANF	0.8048 (0.0000)	0.7297 (0.0000)	0.8143 (0.0000)
	SNF	0.8429 (0.0000)	0.7514 (0.0000)	0.8429 (0.0000)
	PFA	-	-	-
	MVSCO	0.7800 (0.0544)	0.6711 (0.0628)	0.7838 (0.0462)
	LRCMC	**0.8905 (0.0000)**	**0.7922 (0.0000)**	**0.8905 (0.0000)**
WebKB	ANF	0.6798 (0.0000)	0.1718 (0.0000)	0.6946 (0.0000)
	SNF	0.7044 (0.0000)	0.2407 (0.0000)	0.7192 (0.0000)
	PFA	0.7143 (0.0000)	0.3191 (0.0000)	0.8128 (0.0000)
	MVCMO	0.7652 (0.0346)	0.3548 (0.0448)	0.7833 (0.0323)
	LRCMC	**0.8079 (0.0000)**	**0.5081 (0.0000)**	**0.8424 (0.0000)**

- means that the metrics cannot be calculated, the best results have been highlighted in bold.

**Table 3:** Overview of the TCGA datasets.

Datasets	*N*	mRNA Expression	DNA Methylation	miRNA Expression
GBM	213	12,042	1305	534
BIC	105	17,814	23,094	354
LSCC	106	12,042	23,074	352
COAD	92	17,814	23,088	312

**Table 4:** *p* values of survival analysis in Cox log-rank model for different clustering methods of four cancers on The Cancer Genome Atlas (TCGA) datasets.

Methods	GBM	BIC	LSCC	COAD
ANF	5.8 × 10^−4^	3.6 × 10^−4^	8.9 × 10^−3^	9.0 × 10^−3^
SNF	5.0×10^−5^	6.9×10^−4^	7.8 × 10^−3^	1.6 × 10^−3^
PFA	1.8×10^−4^	3.1×10^−4^	1.1 × 10^−2^	2.4 × 10^−2^
MVCMO	1.4×10^−3^	3.5×10^−4^	9.1 × 10^−3^	8.5 × 10^−3^
LRCMC	**1.3 × 10^−5^**	**3.7 × 10^−5^**	**3.8 × 10^−3^**	**1.2 × 10^−3^**

The best results have been highlighted in bold.

**Table 5:** The identified clusters are compared with mRNA-expression-based subtypes and methylation-based subtypes.

Our Cluster	mRNA-Expression-Based Subtypes				Methylation-Based Subtypes	
	Mesenchymal	Classical	Neural	Proneural	G-CLMP	Non-G-CLMP
cluster 1	46	54	27	30	0	155
cluster 2	1	0	1	19	20	1
cluster 3	12	11	7	7	0	37

The values represent the number of patients counted.

**Table 6:** Distribution of genetic variant signatures for the identified clusters.

Our Cluster	*CDKN2A*.del.	*CDKN2B*.del.	*C9orf53*.del.	*MTAP*.del.	*EGFR*.ampl.	*IDH1*
cluster 1	84 (56.4%)	84 (56.4%)	80 (53.7%)	57 (38.3%)	70 (47.0%)	0 (0%)
cluster 2	6 (28.6%)	6 (28.6%)	6 (28.6%)	5 (23.8%)	0 (0%)	10 (66.7%)
cluster 3	24 (68.9%)	23 (62.2%)	24 (68.9%)	21 (56.8%)	19 (51.4%)	0 (0%)

The values indicate the number of variations, and the values in parentheses indicate the frequencies of variations after removing statistical missing. ‘ampl.’: amplification, ‘del.’: deletion.

**Table 7:** GO: BP, KEGG pathway, DO enriched terms for the identified cluster.

ENRICHMENT Analysis	Cluster 1	Cluster 2	Cluster 3
GO:BP enriched terms	1. Epithelial cell differentiation 2. Epithelium development 3. Cell adhesion 4. Biological adhesion 5. cell–cell adhesion	1. Protein targeting to ER 2. Establishment of protein localization to endoplasmic reticulum 3. Protein localization to endoplasmic reticulum 4. Peptide metabolic process 5. Protein targeting protein targeting	1. SRP-dependent cotranslational protein targeting to membrane 2. Cotranslational protein targeting to membrane 3. Nuclear-transcribed mRNA catabolic process, nonsense-mediated decay 4. Protein targeting to ER 5. Establishment of protein localization to endoplasmic reticulum
KEGG enriched pathway terms	1. Cell adhesion molecules (CAMs) 2. Tight junction 3. Pathogenic Escherichia coli infection 4. Leukocyte transendothelial migration	1. Ribosome 2. Protein export	1. Ribosome
DO enriched terms	1. alphaThalassemia 2. Dysfibrinogenemia, congenital 3. Afibrinogenemia, congenital 4. Heinz body anemia	1. Diamond–Blackfan anemia	1. Diamond–Blackfan anemia

We put the GO: BP terms with ranking in the top 5, and KEGG pathway and DO terms with *p*-value less than 1.00E-4 in the table.

## Data Availability

Data are contained within the article and supplementary materials.

## Supplementary Materials

The following are available online at https://www.mdpi.com/article/10.3390/genes12040526/s1, File 1: Source code and datasets.
